# Jenner and Vaccination

**Published:** 1889-07-06

**Authors:** 


### JENNER AND VACCINATION.

THE object of this book* is to make clear to the general reader the steps in the rise and acceptance of Jenner's doctrine and practice of vaccine inoculation. Viewed in the light displayed by the author, Edward Jenner seems little less than a charlatan and an impostor, not only as regards vaccination, but also with reference to other matters which Jenner investigated. The habits of the young cuckoo, for instance, which from our childhood we have been taught to believe removes the eggs and young from the alien nest in which it was hatched, being especially formed by nature for this acrobatic feat. But, says Dr. Creighton, although "Jenner's cuckoo paper contained a few credible and prosaic facts, the greater part of it, and all that part of it which is best remembered, is a tissue of inconsistencies and absurdities." Well! there are a great many other things which we have to unlearn as we get older, and we are prepared, if necessary, to consign the cuckoo story to that ever-increasing limbo of worn-out fallacies. We are not, however, quite prepared to adopt the same course with reference to vaccination, for although, perhaps, as Dr. Crieghton shows, Jenner's reasoning may not always have been accurate and logical, we are, after the lapse of nearly a century, enabled to judge by results. It does not matter that Jenner's attempt to establish a connection between cowpox and grease in horses was a failure, for what concern us most is the query whether vaccination is a failure also? Jenner's assertion was, that if a person were inoculated with the matter from a certain malady affecting the cow, that individual would be proof against smallpox, and Jenner thereupon designated the cow malady as *variola vaccinæ*, or smallpox of the cow. Dr. Creighton objects to this term, and designates Jenner's theory "a cowpox legend." But Mr. Cline, an eminent surgeon of the period, inoculated with cowpox, and found the subject would not take smallpox. According to the author of the book under notice, all the persons lay and professional who took up Jenner's ideas must have been very foolish. Nevertheless, the fact remains that Jenner's views were accepted more or less in all civilised countries. And not only were they accepted, but they were received with acclamation and applause. Of course there were objections even from the first, and on these objections Dr. Creighton lays much stress.

Before the value or otherwise of vaccination can be fully understood, the subject of smallpox should be studied. In Jenner's day it was a horrible and almost universal plague, rendered worse by the practice of inoculating for smallpox, under the impression that if an individual were inoculated from another having the disease slightly, the individual inoculated would also experience only a slight attack. This, however, was not always the case, and every inoculated person became a fresh centre of infection. Although we are not yet free from smallpox, the very fact of vaccine inoculation superseding smallpox inoculation renders the public much less liable to the latter malady. From old records we know that in the last century it was almost exceptional to see a person not marked or in some way disfigured by the disease, whereas in the present day it is exceptional to find people so disfigured, as it is now in some Eastern countries, where vaccination has made no progress, and it would be again in England if vaccination were not pursued. People in England cannot now conceive the effects of unchecked smallpox, and are therefore unable to estimate the exemption secured by vaccination. People in England now live in that security from what was once an universal plague, that they cannot realise the dangers they escape. For smallpox is not only a terrible disease in itself, but is liable to be followed by other maladies, especially by eye affections, oftentimes resulting in partial or total loss of sight.

Statistics might be brought forward proving the value of vaccination, but we refrain from reproducing tabular statements which have so often been published. Suffice it to mention that while the death-rate from smallpox in the London hospitals for a period of fifteen years was 35 per cent., the deaths among those vaccinated and who had taken smallpox afterwards was only ·55 or less than one per cent. In unvaccinated communities smallpox attacks about 90 per cent. of the population, while among the vaccinated only ·60, again less than one per cent. are attacked. In London, before the period of compulsory vaccination, every tenth death was due to smallpox, now only one in every eighty-five is caused by this disease. The protective power of vaccination is well exemplified in hospitals where smallpox patients are received, the nurses employed therein rarely contracting smallpox.

No one ever asserted vaccination to be an infallible preventive of smallpox, for instances of smallpox after vaccination do occur; but, on the other hand, such cases are usually mild in their nature. No one has ever asserted that injurious sequels may not happen after vaccination. A vaccinated child may get erysipelas if this malady happens to be in the locality; but then the child would be as likely to get erysipelas after any accidental scratch on its body as from the scratch made in vaccinating. Again, it seems sufficiently proved that specific disease may be communicated by vaccination. This, however, very seldom results, and must be due to want of care. The vast majority of ailments which have been ignorantly attributed to vaccination have no possible connection with it, although they may have coincided in point of time. It should be recollected that the period after vaccination is ordinarily the time when with the commencement of teething children are most prone to various affections, especially skin eruptions. To attribute all such maladies to vaccination, as is often done, is simply ridiculous. To assert that evil effects have followed vaccination when improperly performed, or when performed from or on diseased children, that smallpox sometimes follows vaccination—advancing these and various other adverse arguments made use of by anti-vaccinationists is simply to assert that vaccination is, like all other human contrivances, not altogether perfect.

As a parliamentary commission of enquiry is about to take place on the subject, we need not pursue it. No doubt the commission will gladly hear both sides of the question. And in this spirit of impartiality they may summon Dr. Charles Creighton as evidence. Doubtless Dr. Creighton, in his book "Jenner and Vaccination," has honestly stated his opinions and conclusions. But has he had sufficient experience practically? The boo shows that as regards the literature of the subject he is fully informed. But this is not all. If we were asked for advice, we should tell Dr. Creighton to place his practice in commission, and to spend a year or two in some Eastern country where there is no vaccination. He need not proceed further than some parts of India. We quote the following account of smallpox in India before the introduction of vaccination (*vide Medical Times and Gazette*, November 27, 1869): "During the recent epidemic, on visiting various towns and villages I was literally surrounded by cases of smallpox. Those suffering from the malady in a minor degree were in the streets—generally children occupied in their accustomed play, or, if too ill, lying neglected on the ground; and these represented but a portion of the whole, the remainder, unable to leave their dwellings, awaiting death in the dark, unventilated structures uses as houses by the native . . . From calculations based on marking the people passing through the gates . . . 10 per cent. have either totally lost the sight of one or both eyes, or have received greater or less permanent injury to other organs. . . . . From special enquiry, I believe nearly 80 per cent. of the population are pitted with smallpox. Unchecked variola is a worse enemy than cholera."

Yet it is this terrible malady which anti-vaccinationists would again permit to stalk unchecked among the people of England.

* "A Strange Chapter of Medical History." By Charles Creighton, M.D. Swan, Sonnenschein, and Co., Paternoster-square. 1889.

